# Supplementary material for: Analysis of PPARγ Signaling Activity in Psoriasis
Source: Int J Mol Sci. 2021 Aug 10;22(16):8603. doi: 10.3390/ijms22168603 (PMC8395241; doi:10.3390/ijms22168603)
Supplement: Supplementary file 1 [file ijms-22-08603-s001.zip › Supplemental materials_Analysis of PPARg signaling activity in psoriasis/Pathway models/Models images and html files/PPARG expressed in cells assotiated with psoriasis/225512.html]

conventional dendritic cell


# CellType conventional dendritic cell

|  |  |
| --- | --- |
| URN | urn:agi-celltype:myeloid%20dendritic%20cell |
| Total Entities | 36 |
| Connectivity | 1013 |
| Name | conventional dendritic cell |

---

|  |  |
| --- | --- |
| ChildConcepts | myeloid dendritic cell differentiation |
|  | CD103+ dendritic cell |
|  | CD141+ dendritic cell |
|  | CD1c dendritic cell |
|  | CD209+ dendritic cell |
|  | CD11c+CD11b+ dendritic cell |
|  | Langerhans cell |
|  | conventional dendritic cell count |
|  | conventional dendritic cell development |
|  | conventional dendritic cell growth |
|  | conventional dendritic cell migration |
|  | conventional dendritic cell function |
|  | conventional dendritic cell survival |
|  | conventional dendritic cell proliferation |
|  | conventional dendritic cell homing |
|  | conventional dendritic cell phenotype |
|  | conventional dendritic cell renewal |
|  | conventional dendritic cell infiltration |
|  | conventional dendritic cell cytotoxicity |
|  | conventional dendritic cell apoptosis |
|  | conventional dendritic cell interaction |
|  | conventional dendritic cell chemotaxis |
|  | conventional dendritic cell population |
|  | conventional dendritic cell formation |
|  | conventional dendritic cell ratio |
|  | conventional dendritic cell motion |
|  | conventional dendritic cell lifespan |
|  | conventional dendritic cell repertoire |
|  | conventional dendritic cell distribution |
|  | conventional dendritic cell viability |
|  | conventional dendritic cell fate |
|  | conventional dendritic cell adhesion |
|  | conventional dendritic cell fate commitment |
|  | conventional dendritic cell polarity |
|  | conventional dendritic cell homeostasis |
|  | conventional dendritic cell transdifferentiation |

---

|  |  |
| --- | --- |
| Pathway | PPARG expressed in cells assotiated with psoriasis |
|  | Neighbors of conventional dendritic cell |

---

|  |  |
| --- | --- |
| MedScan ID | 10004031 |

---

|  |  |
| --- | --- |
| Alias | conventional dendritic cells |
|  | myeloid dendritic cell |
|  | myeloid dendritic cells |
|  | myeloid DC |
|  | conventional dendritic cell |
|  | myeloid DCs |

---
